# Infusion and delivery strategies to maximize the efficacy of CAR-T cell immunotherapy for cancers

**DOI:** 10.1186/s40164-024-00542-2

**Published:** 2024-07-26

**Authors:** Xinyu Gu, Yalan Zhang, Weilin Zhou, Fengling Wang, Feiyang Yan, Haozhan Gao, Wei Wang

**Affiliations:** Department of Biotherapy, State Key Laboratory of Biotherapy and Cancer Center, West China Hospital, Collaborative Innovation Center for Biotherapy, Sichuan University, Chengdu, People’s Republic of China

**Keywords:** CAR-T cells, Immunotherapy, Delivery strategy, Infusion dose

## Abstract

Chimeric antigen receptor (CAR) T-cell therapy has achieved substantial clinical outcomes for tumors, especially for hematological malignancies. However, extending the duration of remission, reduction of relapse for hematological malignancies and improvement of the anti-tumor efficacy for solid tumors are challenges for CAR-T cells immunotherapy. Besides the endeavors to enhance the functionality of CAR-T cell per se, optimization of the infusion and delivery strategies facilitates the breakthrough of the hurdles that limited the efficacy of this cancer immunotherapy. Here, we summarized the infusion and delivery strategies of CAR-T cell therapies under pre-clinical study, clinical trials and on-market status, through which the improvements of safety and efficacy for hematological and solid tumors were analyzed. Of note, novel infusion and delivery strategies, including local-regional infusion, biomaterials bearing the CAR-T cells and multiple infusion technique, overcome many limitations of CAR-T cell therapy. This review provides hints to determine infusion and delivery strategies of CAR-T cell cancer immunotherapy to maximize clinical benefits.

## Introduction

Genetically engineering T cells to express CAR molecules has significantly boosted the advancement of celluar immunotherapy [1]. As a living drug, CAR-T cells are manufactured ex vivo for sufficient expansion and then reinfused into the patient to exert tumor-targted killing activity against tumor cells. CAR-T cell therapy has shifted the paradigm for the treatment of cancer and has become one of the mainstream treatments for refractory and relapsed lymphoma [2]. Currently, six CAR-T cell products for hematologic malignancies have been approved by the American Food and Drug Administration (FDA), achieving impressive clinical outcome in multi-line therapy-refractory patients [3].

Systemic injection of CAR-T cells to patients with hematological tumors has demonstrated feasible and effective [4]. Post-infusion CAR-T cells can circulate throughout the body and dynamically search antigen-specific tumor cells. Upon recognizing target antigen, CAR-T cells are activated to proliferate and exert robust tumor lysis ability [5]. However, poor persistence of CAR-T cells closely correlated with the unsatisfactory outcomes and disease replase in patients receiving CAR-T therapy [6]. Investigators have been endeavoring to develpe various gene-engineering strategies to ameliorate exhaustion and augment the persistence of CAR-T cells, thus improving the duration time of anti-tumor efficacy. From the perspective of clinical administration scheme of CAR-T therapy, the cell-infusion strategy is also one of decisive factors of CAR-T therapeutic effectiveness.

Cytokine release syndrome (CRS) and neurotoxicity are the major side effects in CAR-T cell immunotherapy [7]. High infusion dose of CAR-T cells is identified as one of the main factors causing severe CRS [8, 9]. According to the data disclosed in *Clinical trials.gov*, a varied range of CAR-T cell doses have been investigated to balance the safety and efficacy of CAR-T cell therapy. In addition to considering the total infusion dose, fractional infusion of a total dose provides a strategy to flexibly adjust total CAR-T infusion dose and make the risk of adverse effects more controllable [1, 10].

Based on the success of CAR T-cell therapy in hematological malignancies, researchers have ventured into expanding this therapeutic modality to address solid tumors [11]. However, the concealed location of solid tumors and immunosuppressive microenvironment pose significant barriers, limiting the therapeutic efficacy in treating patients with solid tumors [12, 13]. In terms of CAR-T delivery strategy, the approach differs significantly from that employed in hematological malignancies. Through systemic infusion that is used in treating hematological tumors, CAR-T cells need to circulate, migrate, and break the anatomical barriers to reach the tumor site [14]. During this journey, a portion of CAR-T cells are distributed to different tissues and experience activation-induced mortality within circulation. These conditions can contribute to deficient quantity and quality of CAR-T cells, significantly hampering their anti-tumor activities. Therefore, to overcome these challenges, locoregional delivery strategies such as intraperitoneal injection [15, 16], intrathoracic injection [17], hepatic artery injection, catheter injection [18] have been explored to treat a series of solid tumors. While intravenous (*i.v*) injection of CAR-T cells remains a prevalent method to treat solid tumors [19, 20], which typically requires multiple doses to achieve sufficient effectiveness [21, 22]. Furthermore, innovative adjunctive delivery methods utilizing biomaterials have been developed to boost CAR-T performance [23]. These advanced strategies harness the unique properties of biomaterials to improve CAR-T cell survival, trafficking, and tumor infiltration, thereby potentiating their anti-tumor efficacy. By combining with these interdisciplinary innovations, it is promising to overcome the challenges existing in traditional CAR-T cell delivery mode and unleash the maximal efficacy of CAR-T therapy.

In this review, we mainly focus on the researches regarding infusion dose and delivery strategies of CAR-T cells in treating hematological and solid tumors. Through summarizing the datas and findings disclosed in preclinical and clinical studies, we give insights into choosing proper infusion dose and delivery strategy of CAR-T cells while taking safety and efficacy into account. In addition, we emphasize on novel infusion and delivery techniques including locoregional infusion method, biomaterials-based delivery system and multiple infusion modality, holding great potential to overcome part of the limitations in traditional CAR-T cell therapy. Overall, through a better understanding of the latest CAR-T infusion and delivery strategies, we hope to offer guidance on how to optimize CAR-T cell infusion dose and delivery modality to maximize their clinical benefits for patients with cancer.

## Infusion strategies of CAR-T cell therapy for hematological malignancies

Prior to CAR-T cell infusion, lymphodepletion is a necessity to enable effective and durable therapeutic responses [24]. The common lymphodepletion regimens include cyclophosphamide, fludarabine, bendamustine, azacytidine [25, 26]. In Authority-approved CAR-T cell products CAR-T cell therapies, lymphodepletion chemotherapy typically employs a combination of cyclophosphamide and fludarabine [27]. This regimen can effectively decrease the circulating immune cells, thereby facilitating optimal proliferation and anti-tumor activity of CAR-T cells [28]. We summarized the components, usual doses and schedules of lymphodepletion regimens. At the same time, some common lymphodepletion drugs’ information in clinical trials is also listed (Table 1). After a few days of lymphodepletion, CAR-T cells are intravenously injected into the body and travel in the vascular system. Part of the CAR-T cells can rapidly egress from circulatory system and reside in different tissues, resulting in a quick quantity decrease of circulating CAR-T cells. Upon recognizing the tumor mass, CAR-T cells can be activated to proliferate and alter biodistribution to search for cognate antigens [29]. This long journey can lead to exhaustion, poor persistence and ultimately, unsatisfactory therapeutic efficacy of CAR-T cells. Clinically, to address these challenges, lymphokines/cytokines are often administered to prolong the lifespan and activity of CAR-T cells. Additionally, a wide range of CAR-T cell doses have been explored, spanning one or more orders of magnitude. Next, we focus on summarizing the clinical infusion schemes of CAR-T cells for hematological tumors [30–34].

**Table 1:** Lymphodepletion strategies commonly used in current clinical trials

Lymphodepletion	Dose	Regimens (i.v)	Clinical trial identifier/References
Cyclophosphamide - Fludarabine	**Cy**: 500 mg/m^2^/day **Flu**: 30 mg/m^2^/day	**Cy**-**Flu**: on day-5 to day-3	NCT02348216 NCT03105336 NCT02601313
	**Cy**: 500 mg/m^2^/day for 2 days **Flu**: 30 mg/m^2^/day for 5 days	**Cy**-**Flu**: on day-14 to day − 2	NCT02228096
	**Cy**: 250mg/m^2^/day for 3 days **Flu**: 25 mg/m^2^/day for 3 days	**Cy**-**Flu**: on day-11 to day − 2	NCT02445248
	**Cy**: 300 mg/m^2^/day for 3 days **Flu**: 30 mg/m^2^/day for 3 days	**Cy**-**Flu**: on day-7 to day − 2	NCT02631044
	**Cy**: 900 mg/m^2^/day **Flu**: 25 mg/m^2^/day	**Cy**: on day − 2 **Flu**: on day − 4 to day − 2	NCT02614066
	**Cy**: 300 mg/m^2^/day for 3 days **Flu**: 25 mg/m^2^/day for 3 days	**Cy**-**Flu**: on day-5 to day − 2	NCT03975907
Bendamustine - Fludarabine	**Bendamustine**: 70 mg/m^2^/day for 3 days **Flu**: 30 mg/m^2^/day for 3 days	**Cy**-**Flu**: on day-14 to day − 2	NCT03696784
Bendamustine	90 mg/m^2^/day for 2 days	before CAR-T cells infusion	NCT04516551
Azacitidine - Cyclophosphamide - Fludarabine	**Azacitidine**: 100 mg for 5 days **Cy**: 300 mg/m^2^/day for 3 days **Flu**: 300 mg/m^2^/day for 3 days	**Cy**-**Flu**: on day 3–5 **Azacitidine**: on day 1–5	NCT05797948
Cyclophosphamide	**Cy**: 1.5–3 g/m^2^/day for 1 day	**Cy**: on day − 2	[35]
Busulfan - Fludarabine	**Busulfan**: 3.2 mg/kg /day for 3 days **Flu**: 30 mg/m^2^/day for 5 days	**Busulfan**: on day − 6 to day − 3 **Flu**: on day − 7 to day − 3	[36]

### The infusion dose of CAR-T cells for clinical use

The success of CAR-T cell therapy is evidenced by authority-approved CAR-T cell products for different hematological malignancies. The infusion dose of the CAR-T products for different indications varies (Table 2). According to www.clinical*trials*, we calculated and summarized the disclosed infusion dose or dose range of CAR-T therapies.

**Fig. 1:**
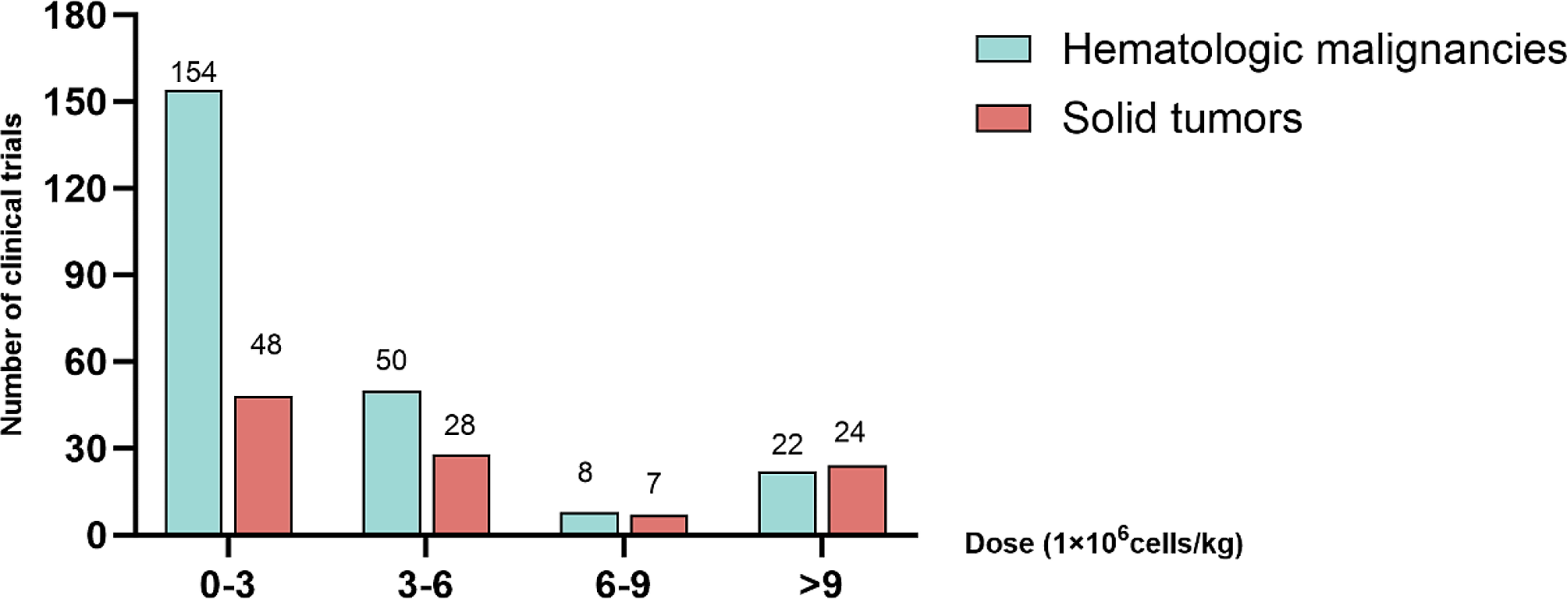
Number of CAR-T clinical trials in different infusion dose intervals. We divide the infusion doses into four dose-intervals. The median value of CAR-T infusion dose range in each clinical trial is calculated and the number of clinical trials in each dose interval is counted. The number of clinical trials is respectively counted in hematologic and solid tumors. The data is summarized according to *clinical trials.gov*

Dose selection is a key point of the success of CAR-T cell therapy. The infusion dose of CAR-T cells is strongly associated with CRS and immune effector cell-associated neurotoxicity syndrome (ICANS) [37]. In the majority of the trials, the infusion doses of CAR-T cells are below 3 × 10^6^ cells/kg for hematological malignancies (Fig. 1). This general threshold of infusion dose may represent as an approximate safe dose, above which may induce adverse effect and uncontrollable outcome [38]. Some studies reported that higher dose levels of CAR-T cells may cause severe toxic effects [39–41]. In a case report, a patient with multiple myeloma was observed the neurotoxicity post high infusion dose of CAR-T cells [42]. In another clinical trial of CD19 CAR-T cells in patients with acute lymphoblastic leukemia, 3 CRS-related deaths observed after CD19 CAR-T cells were infused with a high dose [43]. To mitigate the adverse effect of CAR-T therapy, numerous approaches have been explored in clinical practice. The administration of anti-IL-6 receptor antibody tocilizumab has proven effective in managing CRS, with corticosteroids serving as an additional line of treatment for severe CRS cases [44]. ICANS generally occurs after the symptoms of CRS have subsided. In case of neurologic toxicity, hormonal management is the initial choice due to the inability of monoclonal antibodies rapidly cross the blood-brain barrier. Low-grade ICANS is typically managed by supportive care, whereas severe ICANS is usually treated with corticosteroids [45]. “On-target, off-tumor” also poses a potentially fatal risk in CAR-T therapy. Recently, “suicide genes” including inducible caspase 9 and truncated version of EGFR were incorporated to CAR-T cells to overcome the obstacle [46]. A clinical study published in 2023 demonstrated that the safety performance was enhanced through the use of inducible caspase 9 suicide in patients receiving GD2-CAR-T cells (NCT03373097) [47]. However, irreversible elimination of CAR-T cells by suicide gene prior to eradicate tumor completely might limit clinical efficacy [48]. A potential strategy is designing reversible off/on-switches, which permits CAR-T cell switch between “on” and “off” states. Progress has been made in the administration of some small molecules such as fluorescein isothiocyanate, folate, rimiducid, rapamycin, and proteolysis-targeting chimera compounds [49].

**Table 2:** Authority-approved CAR-T cell products

CAR-T therapy	Target	Cancer type	Dose	Authorized organization
Axicabtagene ciloleucel	CD19	DLBCL and FL	2 × 10^6^cells/kg (maximum of 200 million cells)	FDA/ EMA/ MHLW/ NMPA
Brexucabtagene autoleucel	CD19	r/r MCL	2 × 10^6^cells/kg (maximum of 200 million cells)	FDA/ EMA
	CD19	r/r B-ALL	1 × 10^6^cells/kg (maximum of 100 million cells)	
Tisagenlecleucel	CD19	B-ALL (up to 25 years of age)	0.2-5 × 10^6^cells/kg (≤ 50 kg) 10–250 × 10^6^cells (>50 kg)	FDA/ EMA/ MHLW
	CD19	r/r B-ALL (Adults)	60–600 × 10^6^cells	
Lisocabtagene maraleucel	CD19	r/r LBCL	50–110 × 10^6^cells (1:1 ratio of CAR^+^CD4 and CD8 cells)	FDA/ EMA// MHLW
Idecabtagene vicleucel	BCMA	MM	300–460 × 10^6^cells	FDA/ EMA/ MHLW
Ciltacabtagene autoleucel	BCMA	MM	0.5-1 × 10^6^cells/kg (100 million cells)	FDA/ EMA/ MHLW
Relmacabtagene autoleucel	CD19	r/r LBCL	100 × 10^6^cells	NMPA
Inaticabtagene autoleucel	CD19	r/r B-ALL	0.2 × 10^8^-0.6 × 10^8^cells	NMPA
Zevorcabtagene autoleucel	BCMA	r/r MM	150 × 10^6^cells	NMPA
Equecabtagene autoleucel	BCMA	r/r MM	1 × 10^6^cells/kg	NMPA

EMA: European Medicines Agency; FDA: American Food and Drug Administration; MHLW: Ministry of Health, Labour and Welfare; NMPA: National Medical Products Administration.

The infusion amount of CAR-T cells in different researches varies significantly due to several factors, including the choice of tumor targets, costimulatory domains, and manufacture processes. It has been identified that 4-1BB-incorporated CAR-T cells have superior persistence and less neurological toxicity, compared with CD28 counterparts in clinical tests [50, 51]. Therefore, the choice of CAR-T infusion dose is crucial in determining the efficacy and safety profile of the treatment when it comes to different CAR-T products.

**Fig. 2:**
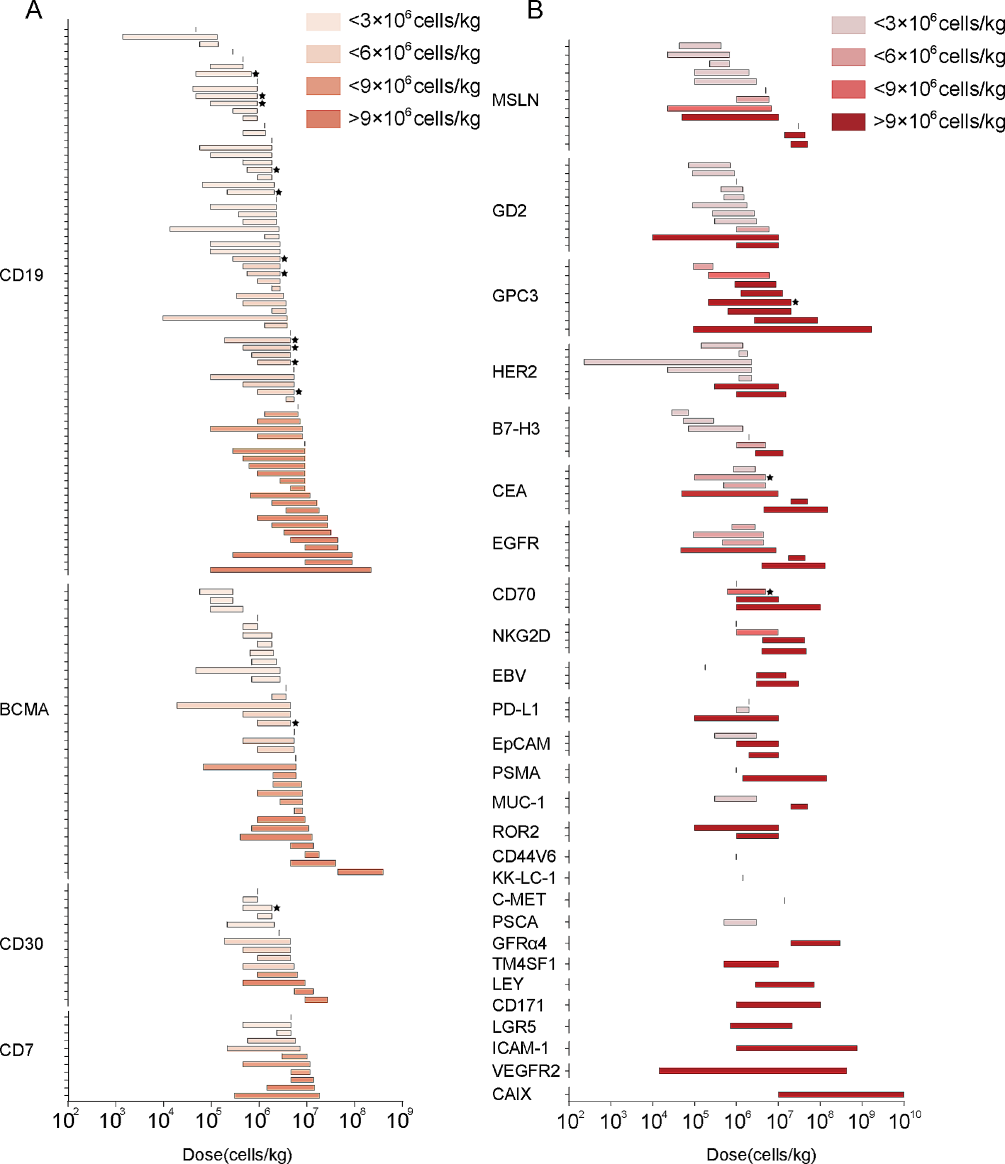
The dose distribution of CAR-T cells on different targets for hematological malignancies and solid tumors in clinical trials. The data is summarized according to *clinical trials.gov.* Each bar represents the CAR-T infusion dose/dose range in a clinical trial(**A**. Summarized clinical trials data for hematological malignancies. **B.** Summarized clinical trials data for solid malignancies) to uniformly compare the infusion dose of CAR-T cells across clinical studies, we normalize the CAR-T dose unit at 10^6^ cells/kg (calculated for 70 kg/patient or 1.6 m^2^/patient if the dose was not flat). The data is ranked in increasing order of CAR-T max dose in each target. (★ represents three or more than three clinical trials adopting the same CAR-T dose/dose range)

CD19-targeted CAR-T cell therapies are the most widely studied in clinical trials. Four CD19-targeted CAR-T products have been approved by FDA for B cell-derived lymphoma and leukemia. The infusion doses of these products are wide-ranging. Taking the tisa-cel for example, adolescents up to 25 years of age receive the low dose (10–250 million CAR-T cells), and the higher dose (60–600 million cells) is suitable for adults (Table 2). However, the lack of transparency in clinical trial reporting, particularly regarding patient information and specific CAR-T cell varieties used, can make it challenging to gain a comprehensive understanding of the dose landscape for CAR-T cell therapies. Nonetheless, by summarizing the dose ranges reported in a mass of studies, we can gain some insights into the general map of CAR-T cell infusion pattern. There are a total of 122 clinical registry trials that have exposed doses of CD19 CAR-T cell therapies. The dose range for the treatment of hematological malignancies is mostly from 1 × 10^6^ to 1 × 10^7^cells/kg (**Fig. 2A**). A positive correlation between therapeutic response and infusion dose levels was reported in some studies [39, 52, 53]. In a phase I trial of CAR-T cell therapy for B-cell lymphoma, patients received a single intravenous infusion at a high dose of 2 × 10^6^ CAR-T cells/kg, the objective response rate(ORR) was 82%, and the complete response rate was 54% [54]. In another phase I trial, bispecific anti-CD20/CD19 CAR T cells for the treatment of recurrent B-cell malignancies were administrated in dose-escalating way, ranging from 2.5 × 10^5^ to 2.5 × 10^6^ cells/kg. The results show the ORR was 100% at the infusion dose of 2.5 × 10^6^ cells/kg (CR was 92% and partial response (PR) was 8%) [55]. It has been reported that CAR-T cell infusion dose has a threshold, beneath which the infusion dose has a positive correlation with the clinical outcome of CAR-T cells. When surpassing this threshold, the clinical response of CAR-T therapy may peak and reach a plateau [38, 56]. Notably, studies involving anti-CD19 CAR-T cells have demonstrated optimal clinical efficacy at doses typically lower than 150 million cells [57–60].

BCMA-targeted CAR-T cell therapies have been approved in the United States for treating multiple myeloma due to their high safety and efficacy. In clinical trials, the dose range of BCMA-targeted CAR-T cells for the treatment of relapsed/refractory multiple myeloma mostly covered the range from 0.5 × 10^6^ cells/kg (NCT03672253) to 5 × 10^6^ cells/kg (NCT04194931) (Fig. 2A). BCMA-targeted CAR-T cell studies may need higher doses [61–63] to achieve optimal clinical efficacy than CD19-targeted CAR-T cells. In a clinical trial, 16 patients received 9 × 10^6^ BCMA-targeted CAR-T cells/kg at the highest dose, attaining 81% ORR and 63% good PR or CR [64].

Transmembrane glycoprotein CD7 is an attractive target in T cell malignancies since it is expressed in over 95% of leukemia and lymphoma produced from T cells [65]. To date, there are 41 clinical trials of CAR-T cell therapies targeting CD7, and most of them cover doses ranging from 0.5 × 10^6^ (NCT04840875) to 6 × 10^6^cells/kg (NCT05127135) (Fig. 2A). A phase I clinical trial was conducted to test genetically modified CD7-targeted allogeneic CAR-T cell therapy in hematologic malignancies. The trial used a dose-escalation design with three levels (level 1: 1 × 10^7^cells/kg; level 2: 2 × 10^7^cells/kg; level 3: 3 × 10^7^cells/kg) to evaluate the safety and tolerability of CD7-targeting CAR-T cells, 81.8% of patients showed objective responses and the CR rate was 63.6% [66]. However, since CD7 is expressed on most T cells, CD7 antigen-specific CAR-T cells can produce severe suicide during preparation. Various techniques including gene editing, protein blockers, and natural selection have been explored to overcome challenges and enhance the capabilities of CD7 CAR-T to lysis T-lymphocyte [67].

CD30, is a type of cell surface glycoprotein that is highly expressed on the surface of Hodgkin’s lymphoma, anaplastic large cell lymphoma, and other lymphoma cells. It is important to note that the expression of CD30 is very low or non-existent on the surface of normal cells and tissues [68]. CAR-T cells targeting CD30 have shown high response rates and low toxicity in patients with relapsed/refractory CD30^+^ hematologic malignancies, particularly in classical Hodgkin lymphoma [69]. To date, an increasing number of CD30 targeting CAR-T cells have been registered in clinical trials, most of which define infusion unit of CAR-T cells by body surface area or total cells. To compare doses across studies, we normalized doses by calculating 70 kg of body weight or 1.6 m^2^ of body-surface area. It was found that CD30 CAR-T dose range mostly covered between 10^6^ to 10^8^cells/kg (Fig. 2A). A research from Baylor College of Medicine and the University of North Carolina showed that autologous CD30 CAR-T cell therapy had a high CR, durability, and a favorable safety profile. Two phase 1/2 trials (NCT02690545 and NCT02917083) involved 41 patients with relapsed/refractory Hodgkin’s lymphoma receiving CD30-targeting CAR-T cells. An expansion cohort of patients at both institutions received the highest dose level of 2 × 10^8^ CAR-T cells/m^2^. The results showed that CD30-targeting CAR-T therapy showed superior efficacy than conventional CAR-T therapy in the treatment of patients with relapsed/refractory Hodgkin lymphoma [70].

The field of CAR-T cell therapy for hematological malignancies has been rapidly expanding, with researchers exploring various novel targets beyond the classical ones like CD19, BCMA, CD7, and CD30 (Fig. 2A). We also provide a valuable overview of the dose ranges being explored for some of these emerging targets in CAR-T cell therapy and listed in Table 3.

**Table 3:** Overview of CAR-T infusion dose of hematological malignancies in clinical studies (not depicted in Fig. 2)

Target	Lymphodepletion	Cancer type	Dose	Clinical trial identifier
CD22	Cy-Flu	B-ALL; DLBCL; FL	0.3;1;3;10 (×10^6^cells/kg)	NCT04088890
CD22	Cy-Flu	B Cell Malignancies; ALL	1 (×10^6^cells/kg)	NCT04088864
CD22	N.A	B Cell Malignancies	0.2–60 (×10^6^cells/kg)	NCT04601181
CD22	N.A	B Cell Malignancies	0.2–60 (×10^6^cells/kg)	NCT05106946
CD22	Cy-Flu	r/r LBCL	1 (×10^6^cells/kg)	NCT05972720
GPRC5D	Cy-Flu	r/r MM	1–6 (×10^6^cells/kg)	NCT05749133
GPRC5D	N.A	r/r MM	3;6;10 (×10^6^cells/kg)	NCT05739188
GPRC5D	N.A	MM	1;3;6 (×10^6^cells/kg)	NCT05016778
GPRC5D	Cy-Flu	r/r MM; PCL	0.5;1;2 (×10^6^cells/kg)	NCT05219721
GPRC5D	Cy-Flu	MM	0.5;1 (×10^6^cells/kg)	NCT03711864
CD33	N.A	AML	3;6;9 (×10^6^cells/kg)	NCT05473221
CD33	Cy-Flu	AML	0.1;0.5;1 (×10^6^cells/kg)	NCT04835519
CD33	Cy-Flu	AML	5 × 10^8^-5 × 10^10^cells	NCT03126864
CD33/CLL1	Cy-Flu	AML	0.5;1;5 (×10^6^cells/kg)	NCT05248685
CD33/CLL1	N.A	AML	1-2.5 (×10^6^cells/kg)	NCT05943314
CD33/CLL1	N.A	AML	3;6;9 (×10^6^cells/kg)	NCT05467254
CD33/CLL1	Cy-Flu	r/r AML	0.5 (×10^6^cells/kg)	NCT05016063
CD5	Cy-Flu	T-ALL	0.5;1;2 (×10^6^cells/kg)	NCT05032599
CD5	Cy-Flu	T-ALL	0.5;1;2 (×10^6^cells/kg)	NCT05487495
CD5	N.A	T-ALL T-NHL	1–5 (×10^6^cells/kg)	NCT04594135
CD5	Cy-Flu	T-ALL T-NHL T-ALL	1;5;10 (×10^7^cells/m^2^)	NCT03081910
CLL-1	N.A	AML	2–8 (×10^6^cells/kg)	NCT05252572
CLL-1	N.A	AML	3;6;9 (×10^6^cells/kg)	NCT05467202
CLL-1	N.A	AML	5–20 (×10^6^cells/kg)	NCT04884984
CLL-1	N.A	AML	1;3;10 (×10^7^cells/m^2^)	NCT04219163
CD123	Cy-Flu	AML	0.5-2 (×10^6^cells/kg)	NCT03672851
CD123	Cy-Flu	BPDCN	6 × 10^8^cells	NCT04109482
CD123	N.A	BPDCN	0.625–6.25 (×10^6^cells/kg)	NCT03203369
CD38	N.A	B-ALL	1–5 (×10^6^cells/kg)	NCT03754764
CD38	N.A	AML	2–8 (×10^6^cells/kg)	NCT05239689
CD38	N.A	AML	5–20 (×10^6^cells/kg)	NCT04351022
CD38/BCMA	N.A	MM	1–5 (×10^6^cells/kg)	NCT03767751
CD4	N.A	r/r T-cell Lymphoma	2–5 (×10^6^cells/kg)	NCT04162340
CD4	Cy-Flu	T-cell lymphoma/Leukemia	0.5;1.5;5;10 (×10^6^cells/kg)	NCT04973527
CD20	N.A	r/r BCL; NHL	1;2;4;8 (×10^6^cells/kg)	NCT04169932
CD20	N.A	r/r BCL	1–20 (×10^6^cells/kg)	NCT03576807
CD20/CD22	Cy-Flu	r/r Lymphoid Malignancies	3–5 (×10^6^cells/kg)	NCT04283006
ADGRE2	N.A	AML	3;6;9 (×10^6^cells/kg)	NCT05463640
CD147	N.A	T-NHL	0.1;0.25;0;5;1;2 (×10^6^ cells/m^2^)	NCT05013372
TRBC1	N.A	TRBC^+^T Cell Lymphoma	25 × 10^6^-9 × 10^8^cells	NCT03590574
FLT3	Cy-Flu	AML	1 × 10^8^;2 × 10^8^;4 × 10^8^cells	NCT05445011
CD7	N.A	CD7^+^Hematologic Diseases	2 × 10^8^cells	NCT05907603
CD138	Cy-Flu	MM	5;10;25;50;100;200 (×10^6^cells/kg)	NCT03672318
CD44V6	Cy-Flu	AML; MM	0.5;1;2 (×10^6^cells/kg)	NCT04097301
SLAMF7	Cy-Flu	MM	0.3–12 (×10^6^cells/kg)	NCT03958656
Igβ	N.A	r/r NHL	1;3;6 (×10^6^cells/kg)	NCT05312476
AGRE2	Cy-Flu	AML	2.5;7.5;22.5;45 × 10^7^cells	NCT05748197
SLAMF7-BCMA	Cy-Flu	MM	0.75-3 (×10^6^cells/kg)	NCT04662099
BAFF	N.A	NHL	1;2;4;8 (×10^7^cells/kg)	NCT05312801
NKG2D	AZA	AML	1;3;10 (×10^8^cells)	NCT03612739
CD19/CD70	N.A	B cell malignancies	1 (×10^6^cells/kg)	NCT05436496
CD70	Cy-Flu	CD70^+^r/r Lymphoma	1;3;10 (×10^6^cells/kg)	NCT05948033

### Dose-split strategy of CAR-T cells infusion

The activation of CAR-T cells is a complex process that can lead to the release of inflammatory cytokines, including interferon and tumor necrosis factor. These substances can trigger the release of additional cytokines from macrophages and monocytes, leading to endothelial damage, CRS and ICANS events [71]. The infusion dose of CAR-T cells, the kinetics of CAR-T cell expansion and tumor burden are the major factors affecting the severity of CRS [8, 9]. Patients with a high tumor burden have been identified with a higher risk of CRS [72, 73]. It has been suggested that CAR-T cell dose fractionation or split dosing can reduce the release of inflammatory cytokines and address the CRS issue [1]. Indeed, in studies using split dosing of CAR-T cells, the incidence of grade 3 or higher CRS can be significantly reduced. Frey NV split the total dose of 500 million CAR-T cells into 10%, 30%, and 60% and infused in the first three days for the treatment of acute lymphocytic leukemia. The results of the study showed that the incidence of CRS in the dose-graded group was significantly lower than that in the low single-dose group [43]. This split dosing strategy has also achieved good clinical results in CD19 CAR-T cells for chronic lymphocytic [26, 74, 75]. In addition to the above dose-split protocol, Xu J et al. divided the total dose into 33%, which was injected on day 0, on day 3, and on day 6 [76]. Some scientists even split the total dose into two (33% and 67%) and infused on the first two days. The results showed that this delivery strategy significantly reduced CRS and improved the safety of CAR-T cell therapy [77, 78]. In patients with high tumor burden, lowering the infusion dose reduces peak cytokine levels and the severity of CRS. However, lowering the dose may also result in an insufficient tumor-lysis, resulting in incomplete clearance of all tumor cells. Thus, the administration of CAR-T cells with a dose-splitting strategy can stagger the rise in cytokine levels, resulting in a lower peak that decreases the severity of CRS.

### Infusion of fresh CAR-T cells vs. cryopreserved CAR-T cells

CAR-T cells are usually cryopreserved to facilitate the completion of rigorous quality control tests and enable flexible infusion schedule based on the patient’s physical condition [79]. However, some studies have found that freshly made CAR-T cells have more potential and advantages compared to cryopreserved ones [80]. Shah et al. observed that patients who received fresh CD20/CD19 tandem bispecific CAR-T cells had increased peak CAR-T cell expansion levels and ORR compared to patients infused with cryopreserved CAR-T cells [55]. Nonetheless, cryopreservation has minimal effect on the fundamental characteristics of CAR-T cells. Studies have consistently demonstrated that the survival rate of resuscitated CAR-T cells following cryopreservation remains high, exceeding 80% [81–84]. In a study published in 2018, the transduction rates of cryo-thawed CAR-T cells from three healthy donors were examined and found no statistically significant differences compared to their pre-cryopreservation counterparts(41.9% vs. 43.5%; 68.3% vs. 69%; 37% vs. 37.3%, *P* > 0.05) [82]. Similar conclusions have been drawn in other studies [83, 84]. Furthermore, cryopreservation has been identified to have negligible effects on the final CAR-T cell composition, as evidenced by the account of CD3 positive cell population(98% ± 2.1% vs. 98% ± 2.4%)and the ration of CD4 and CD8 T cells(2.2 ± 3.9 vs. 2.3 ± 4.0) following resuscitation [83, 85, 86]. Results from a clinical trial comparing the infusion of fresh and cryopreserved targeted CD19 CAR-T cells in Non-Hodgkin’s lymphoma patients showed that the cryopreserved group had a lower rate of acute hematological toxic events compared to the fresh group. One possible reason for the different safety profiles lies in that the quality control parameters of cryopreserved CAR-T cells could remain consistent during transportation from good manufacturing practice facility to the hospitals, but the variant of fresh CAR-T cell parameters was relatively higher [79].

Currently, cryopreserved CAR-T products are still widely employed in clinical trials due to their many advantages over fresh CAR-T cells. Central manufacturing facilities can more easily control the cryopreserved CAR-T cell quality. Additionally, cryopreserved formulations are more cost-effective since they do not require the repeated manufacturing of fresh products for each patient, allowing for more efficient infusion scheduling and patient management [87, 88]. A small number of studies have investigated the effects of cryopreservation on CAR-T cells, more investigations are needed to fully understand the potential impact of this process on cell function and efficacy. Furthermore, based on the convenience and potential of cryopreserved cell product, we should put an emphasis on optimizing the cryopreservation process and to determining the optimal conditions for storing and transporting these cells to ensure the best possible outcomes for patients.

## CAR-T cells delivery strategies of solid tumors

Systematic infusion of CAR-T cells to patients with hematological tumors has achieved encouraging efficacy. Nevertheless, intravenous infusion of CAR-T cells to patients with solid tumors has not replicated the identical success due to the different physical and physiological attributes [89]. Considering these challenges, increasing the intravenous infusion dose and optimizing CAR-T infusion scheme are of significant necessity to ensure the effectiveness of CAR-T cells in solid tumors [90]. Take the hepatic tumor for example, it is desirable to conduct hepatic artery injection to control the volumetric blood flow rate at a low level [91]. Solid tumors grow in concealed locations of the body and form complex tumor microenvironment (TME) such as extracellular matrix (ECM), tumor vasculature, fibroblasts, and immune-suppressive substances, hindering the trafficking and migration of CAR-T cells to solid tumor beds [92]. Therefore, more investigators adopt locoregional infusion methods to deliver CAR-T cells into tumor tissue, which presents as a feasible therapeutic strategy to improve the trafficking, infiltration and efficiency of CAR-T cells. Direct regional injection can avoid the consumption and exhaustion of CAR-T cells during long circulating journey to tumor sites. Due to a more concentrated distribution of CAR-T cells around the tumor bed, off-target and/or dose-related toxicities could be mitigated as well [93, 94].

### The infusion dose of CAR-T cells for clinical use in solid malignancies

Compared to hematological malignancies, the clinical investigation and progression of solid tumors are relatively limited. Investigators have been attempting to target more antigens such as MSLN, HER2, EGFR, GPC3, and Claudin 18.2 to expand the curative potential of CAR-T cells. The tumor antigens targeted by CAR-T products in clinical trials are numerous but few studies have published detailed curative schemes. Since there is little consensus on the number and frequency of CAR-T cells infusion [95], we summarized the infusion dose range of CAR-T cells applied in clinical trials according to the classification of targets. In summary, the infusion dose range of CAR-T cells in clinical trials for solid tumors varies widely, with most doses ranging from 10^5^ to 10^8^ cells per kilogram of body weight (Fig. 2B).

### Regional delivery strategies in different parts of body

**Fig. 3:**
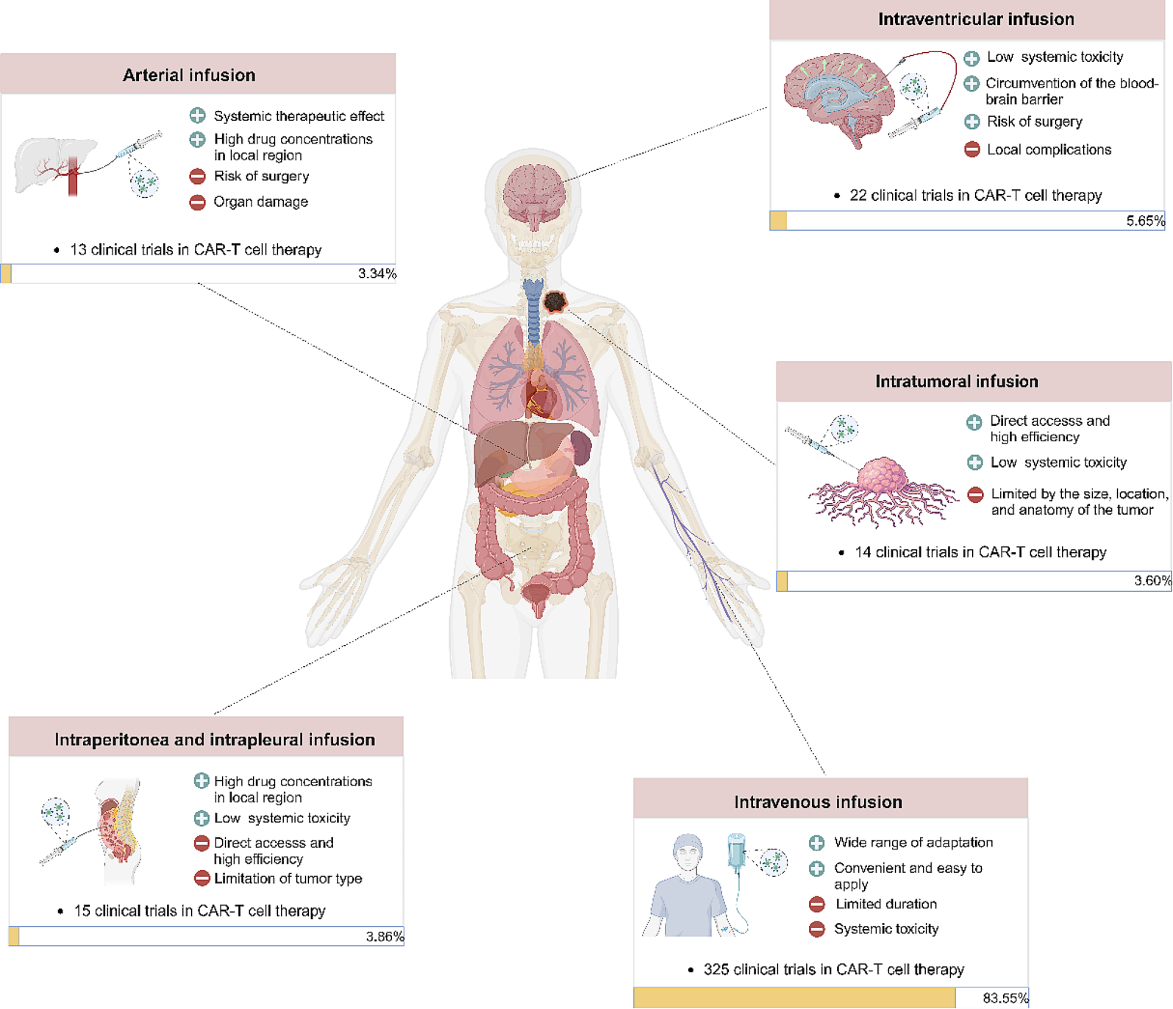
Delivery strategies of CAR-T cells in clinical setting. Intravenous infusion is the major delivery method in treating patients with hematological tumors. Due to the anatomical barrier and TME of solid tumors, multiple locoregional delivery methods have developed for specific tumors. We counted the percent of clinical trials adopting different delivery strategies

Treating solid tumors by CAR-T cell therapy has garnered significant scientific and clinical attention in recent years. Solid tumor clumps tend to be surrounded with abundant tumor-associated fibroblasts and blood vessels [96], forming physical barriers to prevent CAR-T cells from penetrating into the interior of tumor site [97]. In addition, immunosuppressive TME directly impact on the clinical efficacy of CAR-T cell therapy [98]. These factors pose significant challenges for the market translation of CAR-T therapy treating solid tumors. Regional delivery of CAR-T cells has been demonstrated to be safe and feasible in solid tumors [99, 100]. The delivery strategy can promote invasion, proliferation, trafficking, and stimulate functionally sustained systemic immunity. CAR-T cells can be delivered regionally to tumor sites with sustained function. Including intra-tumoral injection, arterial infusion, intraperitoneal injection, and intraventricular injection (Fig. 3).

#### Intratumoral injection

Intratumoral injection can increase CAR-T cell bioavailability inside tumors, enhance the efficacy of immunotherapies and reduce systemic toxicities [101]. Intratumoral injection does not cause direct normal tissue damage compared with resection or radiation. This delivery method is more suitable for visible or palpable tumors, such as melanoma [102, 103]. By the guidance of ultrasound and computed tomography (CT), CAR-T cells can be intratumorally infused in unresectable or medically inoperable tumors [104]. Wang et al. discovered that intratumoral injection of CAR-T cells could eradicate tumors, whereas intravenous injection could only inhibit tumor growth [105]. The injection dose depends on the interstitial pressure and size of the tumor. For refractory and relapsed tumor, multiple intratumoral injections might be needed to stimulate the antitumor immune response [93]. Although repeated punctures on tumor clump can lead to organ damage and the risk of tumor metastasis, clinical outcomes have confirmed the strength of intertumoral injection outweigh its defects in patients whose disease condition have been assessed [106, 107].

To date, there have been 14 clinical trials that have attempted direct intratumoral injection of CAR-T cells (Fig. 3). In a clinical trial of CAR-T cells to treat metastatic breast cancer, patients received a single intratumoral injection of 3 × 10^7^ or 3 × 10^8^ cells. The results showed intratumoral injection of CAR-T cells was well tolerated in all 6 patients. CAR-T mRNA was detectable in peripheral blood and the injected tumor tissue [106]. In a clinical trial published in 2023 for squamous-cell carcinoma of the head and neck, fifteen subjects were treated across five dose cohorts ranging from 1 × 10^7^ to 1 × 10^9^ autologous cells under the guidance of ultrasonography, 60% of subjects obtained disease control, with no treatment-related adverse events above grade 2 were observed [108]. These trials confirmed that intratumoral administration was safe and feasible. This approach could largely reduce systemic toxicities and adverse events since the main immune responses occur locally. Additionally, this approach can combine with other systemic therapies without adding more toxicities [101, 109].

#### Arterial infusion

Intra-arterial delivery is another potential delivery strategy for the regional administration of CAR-T cells [110]. Combining pressure-enabled drug delivery technology with hepatic arterial infusion of CAR-T cells can overcome excessive intra-tumoral pressure and enhance delivery efficiency [111]. Before CAR-T cell infusion, a mapping angiogram was performed via a common femoral artery approach. Next, extrahepatic sites such as the gastroduodenal and right gastric arteries were embolized with microcoils to conduct CAR-T perfusion. Post CAR-T cells injection at a specific speed via a syringe, angiography with a calibrated contrast rate was performed to confirm preserved arterial flow [112]. To date, there have been a limited number of clinical trials that have attempted direct arterial injection of CAR-T cells (Fig. 3). This delivery strategy has been used more frequently in digestive system malignancies. Katz et al. reported their phase I study of local intrahepatic CAR-T cells in the treatment of malignant tumors with liver metastasis, three patients received anti-CEA CAR-T cells through hepatic arterial infusion in dose escalation manner. The results have proved the safety of arterial infusion CAR-T cells [112]. Hepatic arterial infusion of CAR-T cells has also been used in the treatment of colorectal cancer, even receiving a high dose of 1 × 10^10^ CEA CAR-T cells through hepatic arterial infusion, patients with pancreatic cancer did not undergo serious adverse events above grade 3 or on-target/off-target. Compared with the median survival time of 5 months in patients who experienced intravenous injection, the overall survival time of a patient receiving hepatic arterial infusion significantly pronged, up to 23.2 months [113, 114].

#### Intraperitoneal and intrapleural injection

In the past 20 years, intraperitoneal and intrapleural injection of drugs have been mainly used for cancer chemotherapy and achieved good clinical results [115–117]. In recent years, increasing interests have been focused in adopting intraperitoneal and intrapleural delivery strategy to infuse CAR-T cells to solid tumors, showing inspiring efficacy and safety [118–120]. Intraperitoneal infusion have beneficial effect in tumor cells that have unique patterns of spread over the serosal surface [121]. Regional intrapleural and intraperitoneal administration can help increase efficacy and persistence by delivering cells directly into the tumor [122]. In a study that treating epithelial ovarian cancer with ErbB2-targeting CAR-T cells, researchers found that intraperitoneal infusion CAR-T cells offered a safer and more effective strategy than intravenous treatments. The results of this study demonstrated that tumor-bearing mice treated with CAR-T cells by intraperitoneal infusin achieved disease remission and increased survival period compared with intravenous infusion [123]. Additionally, intraperitoneal and intrapleural delivery strategy have also demonstrated potential in clinical stage for treating solid tumors. There have been 16 registered CAR-T clinical trials using intraperitoneal and intrapleural injection to treat solid tumors (Fig. 3), such as malignant pleural mesothelioma (NCT04577326), ovarian cancer (NCT05211557), and pancreatic cancer (NCT03323944). In one phase I trial, a single dose of 1 × 10^6^ CAR-T cells targeting fibroblast-activating protein were delivered to pleural of patients with pleural mesothelioma [124]. The results demonstrated that CAR-T cells indicated an ongoing immune response with a high safety profile in vivo. Intraperitoneal delivery was also utilized in a phase I dose-escalation trial against ovarian cancer and peritoneal mesothelioma. CAR-T cells were injected weekly for 3 weeks (NCT03608618). This study’s preliminary results showed that the treatments were well tolerated, 4 out of 11 patients showed initial stable disease, and 3 patients were in a stable condition for more than 2 months. Combining intravenous with intrapleural injection to deliver CAR-T cells is also a strategy for the treatment of abdominal malignant tumors. In a standard 3 + 3 dose-escalation phase I trial, patients were infused with escalating doses of CAR-T cells from 3 × 10^5^ to 1 × 10^7^ cells/kg to establish the maximum tolerated dose (MTD). All patients will receive 50% of the genetically CAR-T cell dose intravenously, the remaining dose of cells will be administered by intrapleural infusion 3 days later [125].

#### Intraventricular injection

Intraventricular administration of CAR-T cells to target central nervous system (CNS) tumors has shown promising preclinical and early clinical results [22, 90, 126, 127]. In a preclinical study, CAR-T cells were injected intracranially to treat the malignant glioma. Kiwan Kim et al. found that the tumor volume was significantly reduced in tumor-bearing mice and the survival rate of the mice was markedly improved [128]. Infusing CAR-T cells with the assist of intracranial catheter has been demonstrated the efficiency and safety [94, 129]. Nicholas A. Vitanza et al. first reported the efficacy of repeated intracranial B7-H3 CAR-T cells for patients with diffuse intrinsic pontine glioma. The data from this trial suggested the feasibility of repeated intraventricular injection of B7-H3 CAR T-cells, which can induce local immune activation [130]. Clinical trials for the treatment of CNS tumors with different targets can also achieve similar efficacy through intraventricular injection of CAR-T cells. HER2-specific CAR-T cells were repeatedly administered by intraventricular injection to children and young adults with recurrent or refractory HER2-expressing CNS tumors at doses ranging from 1 × 10^7^ to 1 × 10^8^ cells [131]. In March 2023, investigators described the successful intraventricular administration of 1 × 10^5^cells/kg of GD2-Specific 4SCAR-T cells in patients with glioblastoma. 4 of the 8 evaluable patients showed a PR for 3 to 24 months, 1 patient had a stable disease condition for 4 months after infusion [132]. CAR-T cells administered intraventricularly to treat cerebral tumors exhibited faster kinetics, greater potency, and reduced systemic levels of inflammatory cytokines compared with CAR-T cells administered intravenously [90].

In the past 5 years, the number of registered clinical trials exploring locoregional delivery of CAR-T cells in solid tumors has grown considerably. CAR-T cell therapy offers a way to circumvent normal-tissue, on-target, off-tumor toxicity [48]. It allows more concentrated density of CAR-T cells in the solid tumor bed to enhance anti-tumor activity. Significant and durable clinical response have further stimulated the investigators’enthusiam in the advancement of novel regional delivery strategy [99].

## Novel adjunctive delivery strategies of CAR-T cells at the preclinical stage

**Fig. 4:**
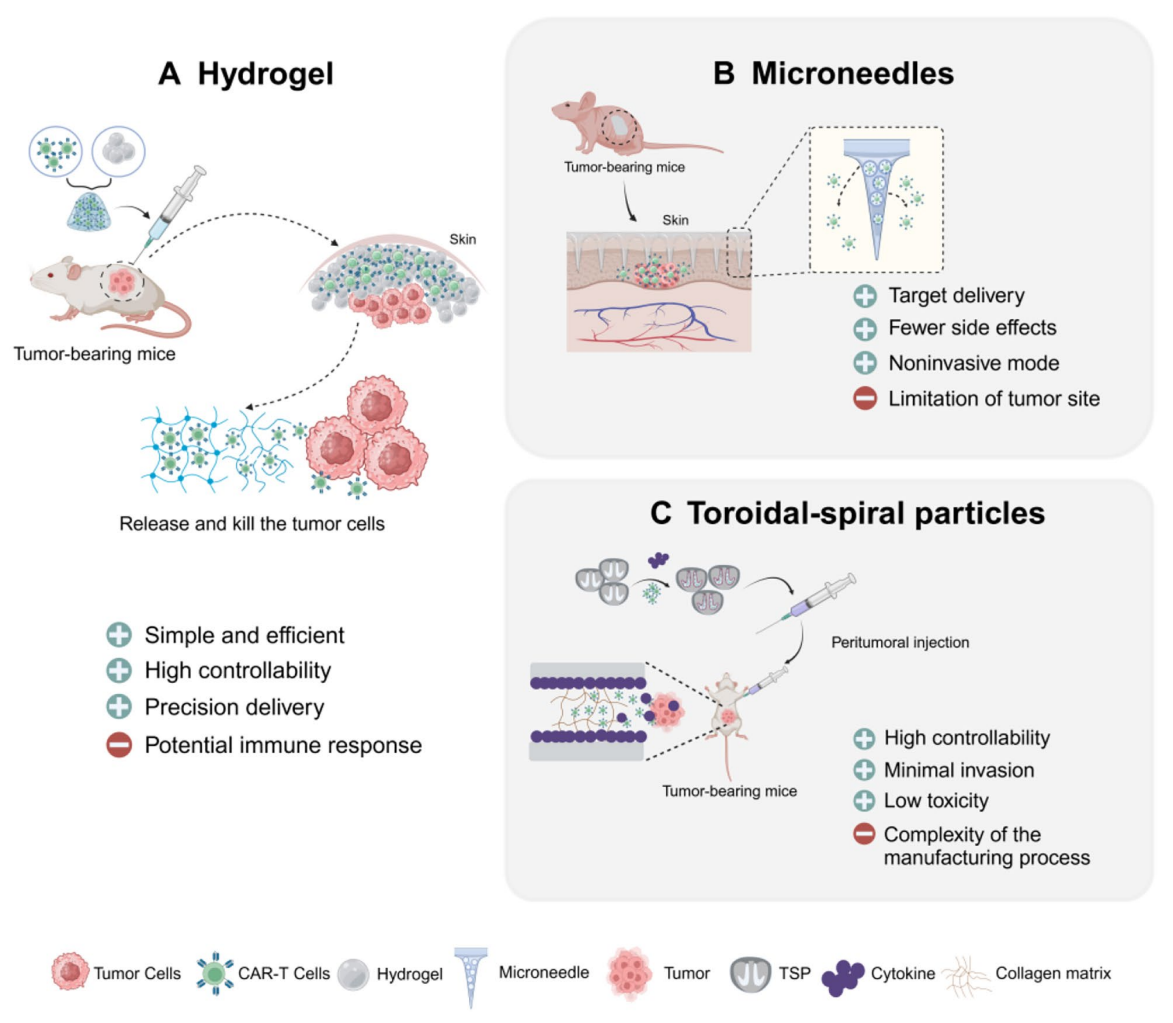
Adjunctive delivery strategies of CAR-T cells in preclinical. The biomaterials, such as hydrogel, microneedles, and toroidal-spiral particles, can load CAR-T cells for scattered seeding in solid tumors, contributing to the improvement of CAR-T therapies. **A** CAR-T cells are wrapped in a special hydrogel, and the hydrogel will continuously release activated CAR-T cells at the site of the solid tumor. **B** CAR-T cells are loaded in the porous structure of microneedles. The needles will release CAR-T cells to kill tumor cells after puncture into tumor tissue. **C** TSP with inner toroidal-spiral channels facilitates CAR-T cell encapsulation, cytokine co-envelope near the surface for controlled release, to stimulate proliferation and activation of CAR-T cells. CAR-T cells are expanded and activated in the device and actively climb out of the collagen matrix toward the tumor cells after peritumoral implantation of the TSPs near the solid tumor

Effective anti-tumor responses require CAR-T cells to be highly activated and persistent at the tumor site [133]. Though locoregional delivery can augment the penetration and viability of CAR-T cells, the lack of sustained cytokine support and harsh immunosuppressive TME can still lead to the exhaustion and dysfunction of CAR-T cells. Biomaterial strategies such as hydrogels, toroidal-spiral particles, implantable biomaterials have been explored to enfold CAR-T cells and immunostimulatory substances, which can greatly enhance the retention and bioactivity of CAR-T cells [134–137] (Fig. 4).

Hydrogel has recently been designed for the local delivery of CAR-T cells to treat solid tumors. Polymer-nanoparticle (PNP) hydrogels compose of water, cellulose polymers found in plants, and biodegradable nanoparticles. The tight mesh structure of hydrogels can load cytokines and CAR-T cells, which forms an enclosed and immune stimulatory environment for CAR-T cells. Post injection of the hydrogel complex through a needle, well-activated CAR-T cells can be slowly released as the hydrogel degrade and distribute in the tumor site. Controlled release of cytokines can support the long-term activation and persistence of CAR-T cells, thus augmenting CAR-T anti-tumor efficacy. Grosskopf found that mice injected with hydrogels containing CAR-T cells and cytokines had better efficacy compared to intravenous injections. Furthermore, the hydrogel significantly degraded in vivo in a few weeks and did not cause any unfavorable inflammatory reactions in the animals [138]. Zhou et al. designed an injectable CAR-T cell local delivery system based on the photo-crosslinked gelatin methacryloyl (GelMA) hydrogels. GelMA hydrogels can not only maintain good solubility but also form a three-dimensional structure by ultraviolet irradiation. It can support the survival and proliferation of CAR-T cells in the TME. GelMA hydrogels also can extend the retention time of CAR-T cells in the tumor site and gradually release them to eliminate tumor cells [139]. Compared to systemically delivered CAR-T cells, hydrogel-based CAR-T cells exhibit higher viability, proliferation, persistence, and anticancer activity. This approach may also prevent the harmful side effects of systemically administered CAR-T cells. These injectable hydrogels may be further developed in the future to allow for more precise regulation of CAR-T cells for long-term treatment lans [140, 141].

Transdermal delivery devices, a minimal and transdermal invasive to deliver drugs by the microneedle patch, can eliminate the possibility of tissue trauma and infection risk associated with injections. Transdermal delivery device makes it possible to conduct a prolonged release of a series of small molecular medications such as galanthamine, insulin, and antibodies [142, 143]. The first cryo-microneedles that could load live cells were created by Xu et al. The therapeutic cells can be delivered to the layer of immune cell-rich epidermis through the microneedles on the skin and can hold a superior persistence and activation. In mice, cells delivered by the cryo-microneedles retained viability and proliferative capability [144]. The depth and distribution of immune cells can be precisely controlled by adjusting the length and cell loading of the microneedles. The loaded cells are successfully delivered by pressing microneedles into the skin, and cryo-microneedle delivery keeps the loaded cells active for a long period. Gu and Li et al. construct a polymeric porous microneedle (PMN) patch to load CAR-T cells.The patch can be implanted in the tumor bed or in the post-surgical resection cavity to delivery CAR-T cells [145]. The microneedle patch offers a multipoint, scattered delivery strategy for CAR-T cells, which can enhance the CAR-T cells infiltration by overcoming physical barriers in solid tumors. More than half of the PMN loaded CAR-T cells were delivered to the tumor within 15 min according to their evaluation of the anticancer effects of CAR-T cells. The investigators also compared the intratumoral distribution of CAR-T cells through intratumoral injection and PMN-mediated delivery in the mice model with WM115 melanoma tumor. Comparing with intratumoral infusion, CAR-T cells delivered via PMN showed more prominent tumor infiltration. Collectively, transdermal administration systems based on microneedles offer a highly modular and efficient approach for CAR-T cell therapy.

Liu et al. designed a biodegradable and biocompatible Toroidal spiral particles (TSP) delivery platform that is universal for different types of lymphocytes. It has strength in high-capacity cell loading, programmable release, high efficacy, low toxicity, and minimally invasive operation. TSP can precisely control the delivery speed of cells, enable in-situ and local delivery of CAR-T cells. The team successfully loaded the MSLN-targeted CAR-T cells into the TSP platform, it triggers an immune response around the tumor and enhances the overall effect of the treatment. Compared to systemic and intratumoral injection, peritumoral delivery of MSLN CAR-T cells using the TSPs resulted in a superior antitumor effect [137].

The application of biomaterials in the adjunctive delivery of CAR-T cells provides a new idea for the treatment of solid tumors. In addition to the three adjunctive delivery strategies mentioned above, an increasing number of materials have been developed such as nitinol thin films, and the alginate scaffold, to enhance the viability, proliferation, persistence, and anti-cancer efficacies of CAR-T cells. Moreover, the CAR-T cells can spread from their implantation sites and circulate to kill distant tumor [146–148].

## Conclusion

CAR-T cell therapy has already changed the therapeutic landscape of hematological malignancies. Enlightened by the extensive preclinical investigation and clinical experience of CAR-T therapy, clinical administration pattern of CAR-T cells plays a critical role in follow-up clinical response and side effect condition. Therefore, a comprehensive knowledge in current infusion dose scheme and delivery strategy of CAR-T cells is of necessity to guide the further therapeutic breakthrough and controllability. Facilitated by the advancement of multi-disciplinary technologies, novel regional CAR-T delivery strategy and biomaterial-based CAR-T delivery methods have been developed to treat refractory solid tumors. The ground-breaking outcomes have confirmed the significance and potential of these innovations, encouraging investigators and clinicians to make effort not only in CAR-T per se, but also in comprehensive consideration of technical combination an clinical practice.

## Data Availability

No datasets were generated or analysed during the current study.

## Abbreviations

ADGRE2: Adhesion G protein-coupled receptor E2
AGRE2: Adhesion G protein-coupled receptor E2 inhibitors
AZA: Azacytidine
AML: Acute myeloid leukemia
BAFF: B-cell activating factor of the TNF family
BCMA: B cell maturation antigen
B-ALL: B cell-acute lymphoblastic leukemia
BPDCN: Blastic plasmacytoid dendritic cell neoplasm
CAR: Chimeric antigen receptor
CAIX: Carbonic anhydrase
CD44V6: CD44 variant domain 6
CEA: Carcinoembryonic antigen
CLL1: C-type lectin-like molecule-1
C-MET: Cellular-mesenchymal epithelial transition factor
CNS: Central nervous system
CR: Complete remissions
CRS: Cytokine release syndrome
CT: Computed tomography
Cy: Cyclophosphamide
DLBCL: Diffuse large B cell lymphoma
ECM: Extracellular matrix
EGFR: Epidermal growth factor receptor
EBV: Epstein-barr virus
EPCAM: Epithelial cell adhesion molecule
FLT3: FMS-like tyrosine kinase
EMA: European Medicines Agency
FDA: American Food and Drug Administration
FL: Follicular lymphoma
Flu: Fludarabine
GD2: Disialoganglioside
GFRα4: GDNF Family Receptor α 4
GPC3: Glypican-3
GPRC5D: G protein-coupled receptor, class C, group 5, member D
GelMA: Gelatin methacryloyl
HER2: Human epidermal growth factor receptor 2
ICAM-1: Intercellular cell adhesion molecule-1
ICANS: Immune effector cell-associated neurotoxicity syndrome
KK-LC-1: Kita-Kyushu lung cancer antigen-1
LEY: Lewis y tetrasaccharide
LGR5: Leucine-rich repeat-containing G-protein coupled receptor 5
MHLW: Ministry of Health, Labour and Welfare
MSLN: Mesothelin
MUC-1: mucin 1
MM: Multiple myeloma
MTD: Maximum tolerated dose
NKG2D: Natural killer group 2, member D
N.A: Not available
NHL: Non-hodgkin lymphoma
NMPA: National Medical Products Administration
ORR: Objective response rate
PD-L1: Programmed cell death 1 ligand 1
PSCA: Prostate stem cell antigen
PSMA: Prostate-specific membrane antigen
PCL: Plasma cell leukemia
PMN: Porous microneedle
PNP: Polymer-nanoparticle
ROR2: Receptor tyrosine kinase like orphan receptor 2 Gene
r/r B-ALL: Relapsed or refractory B cell precursor acute lymphoblastic leukemia
r/r BCL: Relapsed and refractory B Cell Lymphoma
r/r LBCL: Relapsed or refractory large B cell lymphoma
r/r MM: Relapsed and refractory multiple myeloma
r/r MCL: Relapsed and refractory mantle cell lymphoma
r/rAML: Relapsed and refractory acute myeloid leukemia
r/rNHL: Relapsed and refractory non-hodgkin lymphoma
SLAMF7: Signaling lymphocytes activating molecule factor 7
TM4FS1: Transmembrane 4 L six family 1
TRBC1: T cell receptor beta constant 1
T-ALL: T-cell acute lymphoblastic leukemia
TME: Tumor microenvironment
T-NHL: T cell non-hodgkin lymphoma
TSP: Toroidal spiral particles
VEGFR2: Vascular endothelial growth factor receptor 2
